# Exploring immune interactions in triple negative breast cancer: IL-1β inhibition and its therapeutic potential

**DOI:** 10.3389/fgene.2023.1086163

**Published:** 2023-03-29

**Authors:** Brooke E. Wilson, Qiang Shen, David W. Cescon, Michael Reedijk

**Affiliations:** ^1^ Department of Oncology, Queen’s University, Kingston, ON, Canada; ^2^ Division of Cancer Care and Epidemiology, Queen’s Cancer Research Institute, Kingston, ON, Canada; ^3^ Princess Margaret Cancer Centre, University Health Network, Toronto, ON, Canada; ^4^ Division of Medical Oncology & Hematology, Department of Medicine, Princess Margaret Cancer Centre and the University of Toronto, Toronto, ON, Canada; ^5^ Department of Surgical Oncology, University Health Network, Toronto, ON, Canada

**Keywords:** triple negative breast cancer, IL1beta, immunotherapy, tumor microenvironment, inflammasome

## Abstract

Triple negative breast cancer (TNBC) has poor prognosis when compared to other breast cancer subtypes. Despite pre-clinical data supporting an immune targeted approach for TNBCs, immunotherapy has failed to demonstrate the impressive responses seen in other solid tumor malignancies. Additional strategies to modify the tumor immune microenvironment and potentiate response to immunotherapy are needed. In this review, we summarise phase III data supporting the use of immunotherapy for TNBC. We discuss the role of IL-1β in tumorigenesis and summarize pre-clinical data supporting IL-1β inhibition as a potential therapeutic strategy in TNBC. Finally, we present current trials evaluating IL-1β in breast cancer and other solid tumor malignancies and discuss future studies that may provide a strong scientific rationale for the combination of IL-1β and immunotherapy in the neoadjuvant and metastatic setting for people with TNBC.

## Introduction

Breast cancer is the most common malignancy diagnosed in women worldwide (Sung et al., 2021). Triple negative breast cancers (TNBC), defined as tumors lacking expression of the estrogen, progesterone and HER2 receptor, account for approximately 10%–15% of all breast cancers diagnosed in the United States. However, TNBCs are responsible for a relatively large proportion of breast cancer deaths, with 5-year survival being 8%–16% lower than the best prognosis subtype (DeSantis et al., 2019). TNBC is more prevalent in young women, and Black women (19% vs. 9% for White women) (DeSantis et al., 2019), and those with BRCA mutations (Hartman et al., 2012). Emerging research has demonstrated that patients with ER-low disease [1%–9% as per ASCO-CAP criteria (Allison et al., 2020)] have clinical outcomes similar to patients with TNBC (Dieci et al., 2021; Schrodi et al., 2021), raising questions about current classifications systems and treatment paradigms for patients with ER-low disease. Given the poor outcomes associated with this important subtype of breast cancer, there is an unmet need for effective targeted treatments in this population.

TNBCs are heavily infiltrated by immune cells. Multiple studies have demonstrated that high tumor-associated macrophage (TAM) count is inversely related to survival (Schedin et al., 2007; DeNardo et al., 2009; Mahmoud et al., 2012), while high tumor-infiltrating lymphocyte (TIL) count [specifically circulating tumor lymphocytes (CTLs)] is associated with improved survival (Denkert et al., 2010; Loi et al., 2013; Adams et al., 2014; Garcia-Teijido et al., 2016; Savas et al., 2016). In addition to predicting improved survival in TNBC, TIL number predicts increased response to radiotherapy, neoadjuvant and adjuvant chemotherapy, and immunotherapy in a range of tumor types (Stanton et al., 2016; Pruneri et al., 2018). This immune infiltrated microenvironment provides a biological rationale for the treatment of TNBC through modulation of the immune microenvironment with immune checkpoint blockade (ICB) of the programmed death 1 (PD1) or cytotoxic T-lymphocyte-associated protein 4 (CTLA4) pathways (Khalil et al., 2016). In fact, PD-L1 inhibition in combination with chemotherapy has become standard of care for patients with TNBC in the neoadjuvant and metastatic settings. However, even though preclinical data supports an immune-targeted approach for breast cancer, studies to date have failed to demonstrate the impressive results seen in other tumor types such as melanoma and lung cancer, and not all patients with TNBC derive benefit. Ongoing research is needed to identify biomarkers that predict response to immunotherapy in breast cancer. Concurrent strategies to modify the TME to promote response to ICB are needed and one such example is IL-1beta inhibition either alone or in combination with chemotherapy and/or ICB.

Herein, we summarise current clinical evidence supporting the use of ICB in the metastatic and adjuvant/neoadjuvant setting for women with TNBC, discuss the role of IL-1β in tumorigenesis, and summarize pre-clinical and clinical data supporting IL-1β inhibition for TNBC as a novel immune-modulating therapeutic strategy, either alone or in combination with chemotherapy and/or ICB.

## Immunotherapy and TNBC–Metastatic and neoadjuvant

Immunotherapy is now a guideline supported treatment for women with TNBC in both the neoadjuvant and metastatic setting. In the metastatic setting, emerging data suggests immunotherapy is active in selected patients with TNBC. Phase III studies investigating the addition of immunotherapy to standard chemotherapy for first-line metastatic TNBC found improved objective response rates [63% *versus* 55% in Impassion 131 (Miles et al., 2021); 56% vs. 45.9% in Impassion 130 (Schmid et al., 2018); and 41% vs. 35.9% in Keynote-355 (Cortes et al., 2022)]. However, this has not consistently translated into improvements in overall survival in the intention to treat population [mOS 17.2 vs. 15.5 in Keynote 355 (Cortes et al., 2022); 19.2 vs. 22.8 in Impassion 131 (Miles et al., 2021); 21 vs. 18.7 in Impassion 130 (Emens et al., 2021)]. Failure to demonstrate statistically significant improvements in overall survival with atezolizumab in Impassion 130 and the potential detrimental survival effects seen in Impassion 131 led to the withdrawal of approval of atezolizumab for metastatic TNBC by the Food and Drug Administration (FDA). Despite these negative results, subgroup analyses have demonstrated more favourable outcomes in patients with higher levels of PDL1 expression (mOS 25.4 vs. 17.9 months in PDL1 positive Impassion130, 23 vs. 16.1 months in Keynote 355 with CPS>10), but again the findings have been inconsistent (22.1 vs. 28.3 months in Impassion 131).

In phase III studies in the neoadjuvant setting, the addition of immunotherapy to standard chemotherapy for patients with TNBC led to improved pathologic complete response (pCR) (64.8% vs. 51.2% Keynote-522 (Schmid et al., 2020); 58% vs. 41% in Impassion-031; 43.5% vs. 40.8% in NeoTRIP). Some studies have also demonstrated prolonged event free survival [36-month EFS 84.5% vs. 76.8% in Keynote-522 (Schmid et al., 2022)]. As was seen in the metastatic setting, patients with higher PDL1 expression have higher pCR with the addition of immunotherapy, but also have improved response rates to chemotherapy alone, indicating that PDL1 expression may be a prognostic biomarker in the neoadjuvant setting for TNBC.

Although immunotherapy has demonstrated promise as a strategy for women with TNBC, not all patients derive benefit, and additional immune priming strategies to improve ICB response rates in TNBC are needed. Novel therapies capable of modulating immunosuppressive TAMs (Schmid and Varner, 2010; Coussens et al., 2013; Gajewski et al., 2013), which can suppress CTLs through ICB-independent mechanisms (Katakura et al., 2004; Meyer et al., 2014; Noy and Pollard, 2014; Bjoern et al., 2016), are an obvious therapeutic target. IL-1beta is a pleiotropic cytokine capable of recruiting TAMs and generating an immunosuppressive environment, making it an interesting therapeutic target for women with breast cancer.

## The role of IL1β in tumorigenesis in TNBC

**FIGURE 1:**
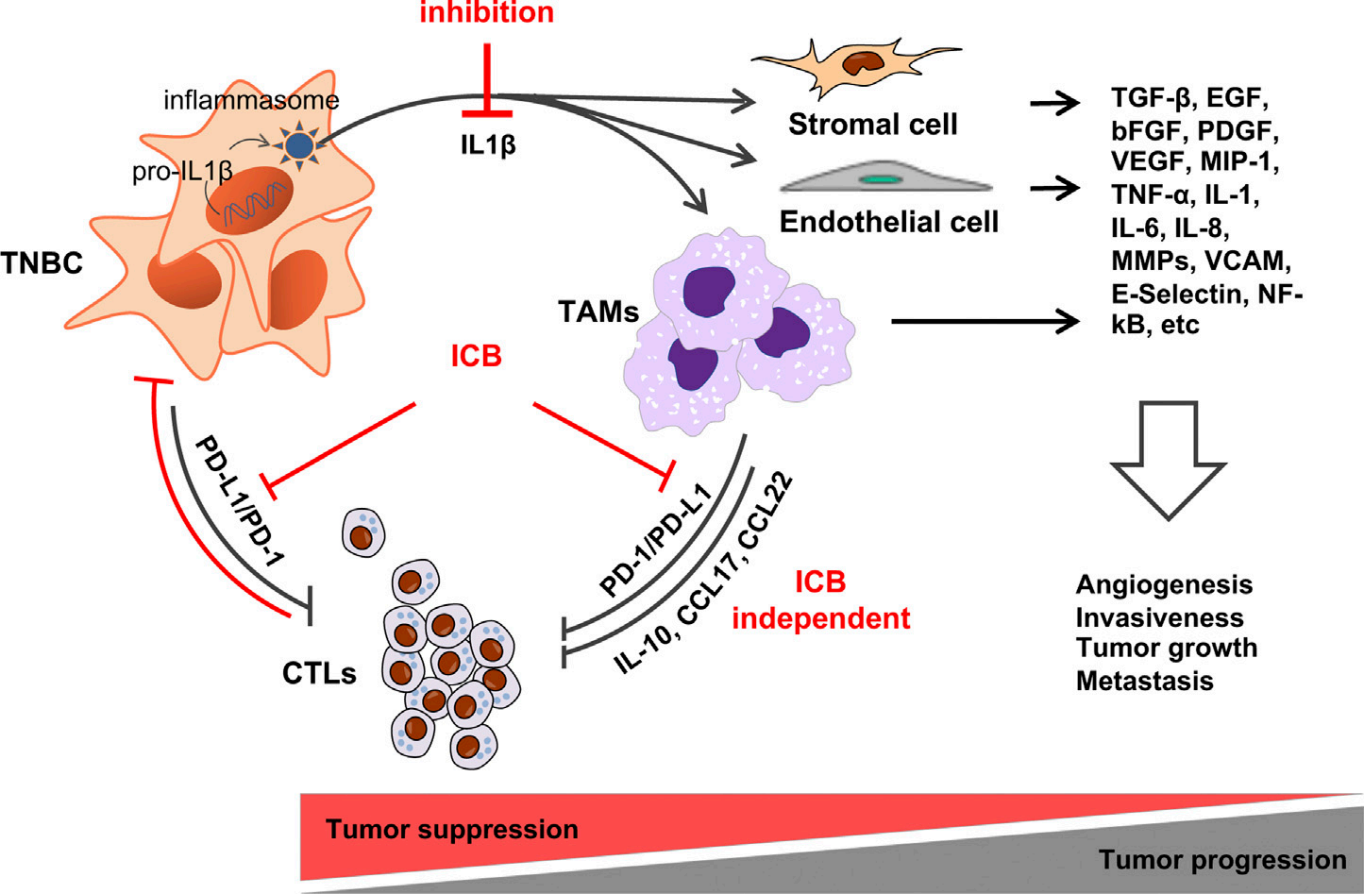
Hypothesis: IL1β-induced TAM recruitment to TNBC promotes tumor progression by inducing immune suppression. TAMs can suppress CTLs via immune checkpoint-dependent and - independent mechanisms making ICB ineffective. By preventing TAM recruitment, and the activation of other cell types within the TME, IL1β inhibition will prevent angiogenesis, tumor cell invasion, metastasis and convert tumors to an ICB-sensitive state. See text for details.

IL-1 has roles in both physiological and pathological states, including angiogenesis, tumor growth and metastases. Studies have shown that IL-1 can be directly produced by cancer cells, or can educate cells within the tumor microenvironment to produce IL-1 (Malik and Kanneganti, 2018), illustrating the complex signalling and interplay between the tumor and surrounding cells. IL-1α is localised in the cytosol and acts within the intracellular environment whereas IL1β is secreted extracellularly and may act on surrounding tissues. Once IL1β binds to its receptor IL-1R1, downstream signalling leads to the activation of NF-kB dependent genes, which in turn can promote cancer growth. Pre-clinical work has found that tumor cell IL1β drives TAM recruitment, immunosuppression, and tumor progression in TNBC (Shen et al., 2017; Jaiswal et al., 2021) (Figure 1). Experimental evidence across most cancer types, supports a tumor-promoting role for IL1β [reviewed (Maund et al., 2013; Ridker et al., 2017; Baker et al., 2019; Gelfo et al., 2020)] making IL1β a target with clear therapeutic potential.

The role of IL1β in growth, invasion and metastases (Guo et al., 2016), stemness and EMT (Oh et al., 2016) has been extensively described. IL1β has an established role in angiogenesis (Krelin et al., 2007) across multiple tumor types. IL1β and vascular endothelial growth factors (VEG-F) are drivers in the establishment and maintenance of cancer-related angiogenesis. Studies have demonstrated that angiogenesis and VEG-F production are IL-1 dependent (Dinarello, 2010), and that IL-1 deficient mice do not induce inflammatory or angiogenic responses (Carmi et al., 2009). IL1β also plays an essential role in the maturation of endothelial precursor cells into endothelial cells by synergistically interacting with VEGF (Voronov et al., 2014). Humans treated with the IL1β antagonist Anakinra for rheumatoid arthritis have reduced numbers of blood vessels in the pannus (Cunnane et al., 2001).

IL1β also plays an important role in tumor cell migration and the establishment of metastatic deposits. IL-1 activates downstream pathways including NF-kB which increases the migratory activity of breast cancer cells, and in turn upregulates CXCL8 under oxygen deprivation (Naldini et al., 2010; Filippi et al., 2015; Storr et al., 2017). The binding of CXCL8 to its receptors CXCR1 and CXCR2 leads to the activation and downstream trafficking of inflammatory mediators, tumor proliferation and breast cancer development (Mishra et al., 2021). In mouse models, it has been shown that by blocking IL-1R, tumor progression and metastasis can be slowed (Holen et al., 2016).

Within the tumor microenvironment, IL-1 has also been shown to modulate anti-tumor activity. IL1β is involved in tumoral recruitment of TAMs and other pro-tumoral inflammatory cells (Kaplanov et al., 2019a). IL-1 secretion can expand and modulate the activities of myeloid derives suppressor cells (MDSC) and downregulate immune surveillance and antitumor immunity (Tu et al., 2008).

Although the majority of published studies suggesting a tumor promoting role for IL-1β, several studies have also demonstrated a tumor inhibiting effect, and the impact of IL1β signalling may vary depending on the cell type and the microenvironment (Dmitrieva-Posocco et al., 2019). For example, breast cancer tumors can elicit a systemic inflammatory response involving IL-1beta expressing innate immune cells which can then migrate and act at distant metastatic lesions. Once these innate immune cells reach the distant metastatic sites, they prevent conversion of tumor cells to a ZEB-1 negative state, thereby limiting epithelioid differentiation and the metastatic cells ability to establish itself. In some pre-clinical models, inhibiting IL-1beta eliminates the differentiation block and drives metastatic colonization (Castaño et al., 2018). However, once the metastatic deposit has been established, IL1beta inhibition did not alter tumor growth.

IL1β secretion requires two-steps. Step 1 (i.e., “priming”) involves the induction of mRNA and protein production of an inactive IL1β pro-protein (pro-IL-1β). Classically this occurs in cells of the innate immune system in response to molecular motifs called ‘pathogen associated molecular patterns’ (PAMPs) that are carried by invading microbes. However, as demonstrated in pre-clinical models, in TNBC priming is driven by aberrant activation of the Notch developmental signaling pathway (Shen et al., 2017). In step 2 of IL1β production (i.e., “cleavage”), in response to PAMPs or ‘danger associated molecular patterns’ (DAMPs), a multiprotein cytosolic complex called the “inflammasome” is assembled (Lopez-Castejon and Brough, 2011; Winsor et al., 2019). With assembly of this complex, pro-caspase-1 (p45) is recruited and activated (Martinon et al., 2002). Activated caspase-1 promotes proteolytic cleavage, maturation, and secretion of IL-1β (Thornberry et al., 1992; Broz and Dixit, 2016). While inflammasomes have traditionally been studied in immune cells, emerging evidence indicates that inflammasome components including caspase-1 are expressed in TNBC cells and correlate with macrophage recruitment (Shen et al., 2017). Thus, TNBCs are uniquely capable of IL1β production due to Notch-induced IL1β priming, and the presence of inflammasome components required for IL1β cleavage, maturation and secretion, making TNBC an excellent candidate for IL1β blockade.

## Pre-clinical models supporting IL1β inhibition for TNBC

Both *in vitro* (Soria et al., 2011; Ma et al., 2012) and *in vivo* (Holen et al., 2016) studies have demonstrated an association between IL-1β expression and metastatic potential for breast cancer. High expression of IL-1β in the primary tumor is associated with disease recurrence at any site for breast cancer, and specifically with bony metastases (Nutter et al., 2014). In mouse models, IL-1 blockade using Anakinra reduced the development and progression of bony metastases from breast cancer (Holen et al., 2016). IL1β knock-out mice treated with orthotopically introduced breast cancer cells showed initial tumor growth followed by subsequent regression, due to the recruitment of alternative inflammatory monocytes in the tumor microenvironment (Kaplanov et al., 2019b). In IL1β deficient mice, increased secretion of IL-12 supports anti-tumor immunity and induces the activation of CD8^+^ T-cell which infiltrate tumors and promote regression (Kaplanov et al., 2019b).

Pre-clinical studies have also shown potential synergistic effects for combining PD1/PD-L1 inhibition with IL-1β inhibition. PD-1 blockade can slow breast cancer tumor growth in some commonly used syngeneic models. However, in one study, combining anti-IL1β antibodies with PD-1 blockade halted tumor progression altogether (Kaplanov et al., 2019b), illustrating that IL1β blockade could facilitate checkpoint inhibition.Clinical data supporting IL1β inhibition in TNBC and other solid tumor malignancies

There are several IL-1β inhibitors already approved by the FDA for a range of rheumatological and autoimmune conditions, including Anakinra, Canakinumab and Rilonacept. IL1β inhibition with either Anakinra or Canakinumab is being explored as a therapeutic strategy in a wide range of solid tumor malignancies, either alone or in combination with chemotherapy, immunotherapy and targeted treatments (Table 1).

**TABLE 1:** Ongoing and complete trials examining IL1β inhibition for solid tumor malignancies (datasource: clinicaltrials.gov).

Study ID	Phase	Setting	Patient population	N	Agent	Intervention	Endpoints	Status	Start	Anticipated completion
Studies including patients with breast cancer										
NCT01802970 (O'Shaughnessy et al., 2016)	I	Metastatic	HER2 negative	11	Anakinra	Anakinra + physicians choice chemotherapy	Safety, CBR, ORR, PFS, IL1 blood transcriptional signatures	Reported	2012	2017
NCT04121442	I/II	Metastatic	Solid tumor, including breast	25	Anakinra	IL1R1 inhibitor Isuanakinra and PD-1/PD-L1 inhibition	DLTs, IL6 reduction, PFS, OS, radiographic response	Results pending	2020	2023
NCT03742349	Ib	Metastatic	TNBC	64	Canakinumab	spartalizumab + LAG525 + canakinumab	AE, SAE, DLT, PFS	Results pending	2019	2022
NCT02900664	1b	Metastatic	Mixed (breast, colon, NSCLC)	283	Canakinumab	PDR001 (checkpoint inhibitor) with Canakinumab		Results pending	2016	2021
**Studies including patients with other solid tumors**										
NCT04942626	I	Stage II/III	Rectal cancer	18	Anakinra	Chemoradiotherapy + Anakinra	Safety, DLT, surgical complications, DFS, OS, local recurrence	Ongoing	2021	2026
NCT02090101	II	Metastatic	Colon cancer	32	Anakinra	LV5FU2 Bevacizumab Plus Anakinra	RR at 2 months, OS, tumor control rate, safety	Complete, results pending	2014	2017
NCT00072111	I	Metastatic	Solid tumor with IL1 expression	NA	Anakinra	Anakinra	DLT and safety	Complete, results pending	2003	unknown
NCT01624766	I	Metastatic	Mixed tumor types	57	Anakinra	Anakinra + Everolimus or Denosumab + Everolimus	MTD, AE	Completed, results pending	2012	2021
NCT02021422	I	metastatic	Pancreas	13	Anakinra	Anakinra + mFOLFIRINOX	AE, SAE, OS, immune modulation	Active, not recruiting	2013	2017
NCT02550327	I	Metastatic	Pancreas	20	Anakinra	Gemcitbine, nab-paclitaxel, cisplatin and anakinra	DFS, OS, QOL, safety	completed	2016	2021
NCT04926467	II	Resectable	Pancreas	20	Anakinra	Chemotherapy + anakinra	Ca 19.9 trends	Not yet recruiting	2021	2026
**Studies examining canakinumab**										
NCT04905316 (CHORUS)	I/II	Unresectable	NSCLC	24	Canakinumab	Canakinumab + chemoradiotherapy + durvalumab	PFS, pneumonitis	Recruiting	2021	2024
NCT03447769 (CANOPY-A) (Garon et al., 2022)	III	Adjuvant	NSCLC	1382	Canakinumab	Canakinumab vs. placebo	DFS, OS	Complete	2018	2026
NCT04789681 (Can-Prevent-Lung)	II	prevention	NSCLC	50	Canakinumab	Canakinumab	Regression of indeterminate pulmonary nodules, lung cancer free survival	Ongoing	2021	2023
NCT03626545 (CANOPY-2) (Paz-Ares et al., 2021)	III	Metastatic	NSCLC	245	Canakinumab	Canakinumab + docetaxel vs. placebo + docetaxel	Safety, OS, ORR, PFS	Ongoing	2019	2021
NCT04581343	Ib	Metastatic	Pancreas	10	Canakinumab	Canakinumab, spartalizumab, nab-paclitaxel, gemcitabine	Safety, dosing	Ongoing	2020	2023
NCT04229004	III	Metastatic	Pancreas	825	Canakinumab	Canakinumab, spartalizumab, nab-paclitaxel, gemcitabine (multiarm trial, this is one arm	OS, PFS, ECOG, ORR	Ongoing	2020	2024
NCT03064854	Ib	Metastatic	NSCLC	111	Canakinumab	Multiarm including PDR001 + canakinumab + cisplatin + pemetrexed	DLT, ORR, PFS	Terminated early		
NCT03484923	II	Metastatic	Melanoma	196	Canakinumab	Multiarm, including canakinumab + spartazliumab	ORR, duration of response, OS, PFS	Ongoing	2018	2022
NCT03968419 (Garrido et al., 2021) (CANOPY-N)	II	Neoadjuvant	NSCLC	88	Canakinumab	Canakinumab + Pembrolizumab	Major pathologic response, antidrug antibodies	Results pending	2019	2022
NCT03631199 (CANOPY-1)	III	Metastatic	NSCLC	673	Canakinumab	Canakinumab + Pembrolizumab	Safety, PFS, OS	Results pending	2019	2022
NCT04028245	I	Localized	RCC	14	Canakinumab	canakinumab plus spartalizumab	% patients proceeding to radical nephrectomy	Results pending	2019	2023

DLT, dose limiting toxicity; RCC, renal cell carcinoma; OS, overall survival; ORR, objective response rate; PFS, progression free survival; NSCLC, non,-small cell lung cancer; TNBC, triple negative breast cancer; DFS, disease free survival; SAE, serious adverse event; AE, adverse event; CBR, clinical benefit ratio; QOL, quality of life; RR-recurrence rate.

To our knowledge, only one reported early phase trial has investigated IL1β inhibition for women with breast cancer. NCT01802970 (O'Shaughnessy et al., 2016) enrolled 11 patients with HER2 negative metastatic breast cancer and treated them with daily administration of Anakinra in addition to physician’s choice chemotherapy. The combination was well tolerated, with the predominant toxicity of Anakinra being injection site reactions (O'Shaughnessy et al., 2016). Four patients had reduction in tumor volume, 4 had stable disease, and 3 had progressive disease, for an overall response rate of 36%. Two patients stopped Anakinra for injection site reactions. Several other studies in breast cancer examining IL-1β inhibition either alone or in combination with chemotherapy or immunotherapy in the metastatic setting are ongoing (NCT04121442, NCT03742349, NCT02900664).

In addition to the early phase data supporting the role of IL1β inhibition in solid tumor malignancies, the phase III CANTOS trial randomised 10,061 patients with heart failure to the IL1β inhibitor canakinumab (Ilaris^®^) or placebo (Ridker et al., 2017), and found a greater than 50% reduction in the risk of death from all cancers. Although the primary endpoint of this study was non-fatal myocardial infarction, non-fatal stroke or cardiovascular death, and the study was not powered to investigate differences in cancer outcomes, these findings support pre-clinical data on the potential role of IL1β inhibition for cancer control. However, subsequent phase III studies in lung cancer including CANOPY-A and CANOPY-2 have failed to demonstrated improvements in progression-free or overall survival (Paz-Ares et al., 2021; Garon et al., 2022), raising questions about this therapeutic strategy in unselected patients. More recent data has identified a potential mechanism for the recognised link between air pollution and lung cancer. Swanton et al. (2022) found that exposure to 2.5 µm particulate matter led to an influx of macrophages and inflammatory mediators including IL-1β which induces a progenitor-like state in the lung epithelium harbouring mutant EGFR, promoting tumor formation. Together, these findings provide compelling evidence of a role for IL-1β in tumorigenesis, and highlight the ongoing work needed to identify valid biomarkers in this patient population in order harness the therapeutic potential of IL-1β blockade for patients with breast cancer and other solid tumor malignancies.

## Future directions and ongoing research

Ongoing work *in vivo* is required to better understand the immunomodulatory effects of IL-1β inhibition in patients with TNBC, and the potential synergism with ICB and other immunomodulatory drugs. In an upcoming study, we will evaluate IL-1β blockade in the neoadjuvant setting for women with early TNBC, examining changes to key immune biomarkers of the tumor microenvironment (including TILs, TAMs, NK cells, IL-1β and inflammasome expression) using paired samples pre and post anti-IL-1β therapy. This in-depth analysis using multiplex immunohistochemistry, high-dimensional mass cytometry, and T and B-cell repertoire analysis will provide much needed granular data on the immunomodulatory effects of IL-1β blockade for women with TNBC and provide a strong scientific rationale for future studies examining the therapeutic potential of the combination of IL-1β blockade and immunotherapy in the neoadjuvant and metastatic setting.
